# Endocrine Disorders in Children with Brain Tumors: At Diagnosis, after Surgery, Radiotherapy and Chemotherapy

**DOI:** 10.3390/children9111617

**Published:** 2022-10-25

**Authors:** Fabien Claude, Graziamaria Ubertini, Gabor Szinnai

**Affiliations:** 1 Department of Pediatric Endocrinology and Diabetology, University Children’s Hospital Basel, University of Basel, 4056 Basel, Switzerland; 2 Department of Pediatric Endocrinology, Bambino Gesù Children’s Hospital, 00165 Rome, Italy; 3 Department of Clinical Research, University Hospital Basel, University of Basel, 4056 Basel, Switzerland

**Keywords:** brain tumor, pituitary gland, endocrine deficits, diabetes insipidus, long-term follow-up

## Abstract

Introduction: Brain tumors are the second most frequent type of all pediatric malignancies. Depending on their localization, patients with brain tumors may present neurological or ophthalmological symptoms, but also weight anomalies and endocrine disorders ranging from growth hormone deficiency, anomalies of puberty, diabetes insipidus to panhypopituitarism. Immediately at diagnosis, all patients with brain tumors require a complete assessment of the hypothalamic–pituitary function in order to address eventual endocrine disorders. Moreover, children and adolescents undergoing brain surgery must receive peri- and postoperative hydrocortisone stress therapy. Post-operative disorders of water homeostasis are frequent, ranging from transient diabetes insipidus, as well as syndrome of inappropriate antidiuretic hormone secretion to persistent diabetes insipidus. Late endocrine disorders may result from surgery near or within the hypothalamic–pituitary region. Pituitary deficits are frequent after radiotherapy, especially growth hormone deficiency. Thyroid nodules or secondary thyroid cancers may arise years after radiotherapy. Gonadal dysfunction is frequent after chemotherapy especially with alkylating agents. Conclusion: Early detection and treatment of specific endocrine disorders at diagnosis, perioperatively, and during long-term follow-up result in improved general and metabolic health and quality of life.

## 1. Introduction

Brain tumors are the second most frequent type of all pediatric malignancies and the most common cause of solid tumors in children and adolescents [1,2,3]. They are the leading cause of pediatric oncological morbidity and mortality [4,5]. The incidence rate of malignant tumors is highest in early childhood, while non-malignant tumors show a continuous rise until adolescence [6,7]. Malignant brain tumor incidence increased in children and adolescents slightly by 0.5 to 0.7% per year from 2008 to 2017 in the United States [7]. In this review, we will outline the endocrine disorders associated with pediatric brain tumors at diagnosis or as a consequence of the therapeutic modalities.

Pediatric brain tumors may be classified according to their cellular origin [4,8,9,10]. Insights into genetic pathogenesis by advances in molecular biology have led to the identification of different subsets of tumors with distinct treatment options and prognoses [5,9].

Gliomas (low and high grades together) are the most frequent type, especially in older patients. They are then followed by tumors from embryonal precursors (medulloblastoma, atypical teratoid/rhabdoid tumors), particularly in young children [1,4,9,11]. Ependymal tumors are the third most frequent type of brain tumors. Choroid plexus tumors and germ cells tumors are less common in pediatrics. However, germ cells tumors frequently arise in the region of the pineal gland and may, thus, compromise the function of the hypothalamic–pituitary axis [1,4,9,12].

The localization of brain tumors is, thus, important to consider in relation to the risk of eventual neurological or ophthalmological symptoms and endocrine disorders [13]. According to their localization below or above the tentorium, brain tumors can be divided into the following categories:

- *Infratentorial tumors* involving the cerebellum, brain stem, and fourth ventricle;
- *Supratentorial tumors* involving the hypothalamus, thalamic nuclei, corpus callosum, basal ganglia, lateral ventricles, and cerebral hemispheres.

Supratentorial tumors are common in patients under the age of 4 years or above 10 years, while infratentorial tumors are frequent in patients between the age of 4 years and 10 years [4]. Supratentorial tumors (especially if located in the diencephalon, also called central tumors) or their treatment may directly compromise the hypothalamic–pituitary axis, leading to endocrine disorders.

## 2. Brain Tumors and Endocrine Manifestations

Patients with pediatric brain tumors usually present with neurological symptoms, such as a headache, vomiting due to increased intracranial pressure, abnormal gait or coordination, papilledema, seizures, or ophthalmological problems [12,13,14,15,16].

As described above, brain malignancies can be divided into infra- and supratentorial tumors. However, neurological and ophthalmological symptoms can be more finely defined according to five localization subgroups [13], as follows:

- *Infratentorial tumors* are divided into (1) brain stem tumors, (2) tumors of the posterior fossa and (3) spinal cord tumors;
- *Supratentorial tumors* are divided into (4) supratentorial tumors (except diencephalic tumors) and (5) central tumors (of the diencephalon).

In general, there is often a long delay between the onset of non-specific symptoms and the diagnosis of brain tumors. In the United Kingdom, a public and professional awareness campaign for caregivers and medical personal was started in 2011 to accelerate brain tumor diagnosis in children (www.headsmart.org.uk, accessed on 8 July 2022, see Table 1) [17]. The authors observed a reduction in the time to diagnosis of pediatric brain tumors (defined as the time between the first symptoms and the definitive diagnosis) from 14.4 weeks in 2006 [18] and 9.1 weeks in 2011 before the campaign to 6.7 weeks in 2013 after its implementation [18]. There were differences among age groups and tumor localization; the longest median time to diagnosis was observed for centrally localized tumors (10.5 weeks) compared to tumors in the cerebellum (7.4 weeks), cerebral hemisphere (6.7 weeks), and brainstem (5.4 weeks) [18]. The time to diagnosis was longest in patients aged 12–18 years (12.3 weeks) compared to those aged 0–5 years (6.0 weeks) and 5–11 years (8.0 weeks) [16,17,18].

Endocrine manifestations are mostly caused by central tumors of the third ventricle, tectum, pineal gland, pituitary gland, thalamus, hypothalamus, optic pathway, and basal ganglia [13,19,20].

Endocrine disorders usually present together with neurological symptoms. However, they may precede the onset of visual disturbances [19]. In a retrospective study of *n* = 176 patients with hypothalamic–pituitary lesions, 50% presented with neurological signs, 38% with neuroophthalmological signs, and 34% with any endocrine disorders [19]. Neuroophthalmological symptoms led to the diagnosis of a brain tumor in a subgroup of *n* = 122 patients with a median interval between the onset of symptoms and diagnosis of three months [19]. As per a critical review of these 122 patients’ charts with hypothalamic–pituitary lesions and neuroophthalmological symptoms, abnormal BMI and growth velocity were present in 63% and 66% of cases, respectively [19]. They were evident at a median of 2.5 years and 1.1 years prior to diagnosis of a brain tumor, respectively [19]. Precocious puberty occurred in 6% and diabetes insipidus was observed in 8% of patients, 1.7 years and 0.8 years before the diagnosis of a brain tumor, respectively [19]. In contrast, the role of brain tumors for abnormal BMI at diagnosis has recently been questioned by a large Dutch cohort. They found no association between BMI and pituitary deficiencies in *n* = 685 patients with brain tumor at diagnosis [21]. However the same authors described in the same cohort that 33.1% of patients developed significant weight gain, overweight, or obesity during follow-up [22].

However, physicians should maintain a high level of suspicion in the context of such initially often subtle auxological and clinical abnormalities [16,19]. Based on signs and symptoms suggestive for brain tumors, a decision support tool has been developed in the United Kingdom integrating the wide spectrum of possible initial signs (based on national guidelines for the diagnosis of brain tumors in children), which provides guidance on further management (e.g., MRI), including endocrine disorders, such as delayed puberty, abnormal growth, and diabetes insipidus (www.headsmart.org.uk, accessed on 8 July 2022, see Table 1) [16,17,18].

Furthermore, specific endocrine deficiencies may be suggestive of specific brain tumors; precocious puberty is a classical sign of hamartomas, mostly without ophthalmological symptoms [19]. Diabetes insipidus often precedes the diagnosis of brain tumors [23]. Moreover, diabetes insipidus is often present years before the diagnosis of Langerhans cell histiocytosis [24,25,26].

The endocrine work-up includes the basal assessment of all of the hypothalamic–pituitary axis and the posterior pituitary function, including electrolytes, plasma and urine osmolarity. In case of documented altered function [27], further investigations are mandatory.

## 3. Peri and Post-Surgery Endocrine Complications and Their Management

Surgery remains the mainstay treatment for pediatric brain tumors [27,28], including minimally invasive techniques, such as transsphenoidal resection for masses of the pituitary and the diencephalon [29]. Transient complications, such as infection, cerebrospinal fluid leak, bleeding, metabolic disturbances, brain swelling, and focal deficits may occur [28,29].

### 3.1. Alterations of Water Homeostasis

Patients with pituitary or diencephalon surgery often develop alterations of water homeostasis with polyuria and electrolyte disturbances, such as hyper- or hyponatremia, which should be promptly identified in order to avoid neurological sequelae [29,30,31,32,33,34].

Central diabetes insipidus can occur as a transient or permanent form. Transient central diabetes insipidus occurs 1–2 days post-surgery and lasts on average 5–7 days [30,33,34,35]. Permanent diabetes insipidus usually develops in a triphasic way [30,33,34,35]. Due to a temporary alteration of antidiuretic hormone (ADH) secretion caused by edema, patients may demonstrate hypotonic polyuria during the first phase, potentially leading to hypernatremic dehydration [30,33,34,35]. During the second phase, degenerating hypothalamic or posterior pituitary cells release an inadequate quantity of ADH in the context of a syndrome of inappropriate ADH secretion lasting 2–14 days, and leading to hypertonic oliguria and hyponatremic fluid overload [30,33,34,35]. Finally, permanent diabetes insipidus results from irreversible damage of the posterior pituitary gland [30,33,34,35]. Post-neurosurgical measurement of copeptin—a surrogate marker of ADH—has been shown to predict post-surgical diabetes insipidus in adults with high sensitivity and specificity [36]. Systematic data are not available in pediatric patients, but copeptin measurements after surgery may help to follow the different phases of water metabolism disturbances.

Hypernatremia and polyuria due to diabetes insipidus requires treatment with desmopressin, an ADH analogue with prolonged antidiuretic and reduced vasoactive effect. The starting dose needs to be cautiously individualized and titrated with the lowest possible dose until stable euvolemic status and normal sodium levels throughout the day are achieved [33,37,38,39,40]. As central diabetes insipidus can be transient, at first only a single and low dose of desmopressin should be administered, and further doses are only given if there is evidence for persisting or recurring polyuria (breakthrough polyuria) [33]. Overdosing of desmopressin will cause water intoxication with anuria and hyponatremia [33,39,40].

Desmopressin maintenance doses for persistent central diabetes insipidus according to the literature range from [33,35,37,38,39,40], as follows:

- Tablets—25–500 mcg/day in three doses;
- Intranasal—2.5–20 mcg/day in one–three doses;
- Sublingual—1–4 mcg/kg/day.

Higher doses are rarely needed [33,35,37,38,39,40].

Hyponatremia and hypervolemia due to the syndrome of inappropriate ADH secretion requires water restriction as a mainstay of treatment [33,34,35,41,42]. The syndrome of inappropriate ADH secretion is usually transient [33,34,35,41,42]. There are no trials in the pediatric population regarding the use of the new ADH antagonists, which competitively interfere with ADH binding to the vasopressin 2 receptor in the kidney [43].

Hyponatremia in the context of the syndrome of inappropriate ADH secretion needs to be differentiated from cerebral salt-wasting and the adrenal crisis (see Table 2) [29,33,34,35,42]. Cerebral salt wasting is a consequence of inappropriate secretion of natriuretic peptides (pro-atrial natriuretic peptide, pro-brain natriuretic peptide) [29,33,34,35,42]. While hyponatremia due to water retention is typical in the syndrome of inappropriate ADH secretion, cerebral salt wasting is characterized by dehydration and volume depletion due to natriuresis and diuresis [29,33,34,35,42]. As a consequence, treatments are different, as the syndrome of inappropriate ADH secretion requires water restriction, while rehydration with sodium supplementation is fundamental in cerebral salt wasting [29,33,34,35,42]. Both diabetes insipidus and syndrome of inappropriate ADH secretion may be complicated by cerebral salt wasting [44,45].

Finally, adrenal crisis with cortisol deficiency is characterized by hyponatremia, hypoglycemia, hypovolemia, tachycardia, and shock, and always needs to be considered in the context of post-neurosurgical patients with hyponatremia [33].

### 3.2. Central Hypocortisolism and Adrenal Crisis

The preoperative assessment of the corticotropic axis is fundamental. At diagnosis, patients presenting a reduced cortisol secretion must be treated with a basal oral hydrocortisone substitution until surgery. According to the current literature, 8–10 mg/m^2^/day in three divided doses is suggested for adrenal insufficiency [46,47].

Hydrocortisone stress therapy must be given intravenously before, during, and after brain surgery in all children and adolescents—even those with normal cortisol secretion. The United Kingdom and Ireland guidelines recommend 2 mg/kg intravenously at induction and every four hours thereafter, or continuous intravenous infusion according to body weight (up to 10 kg body weight, 25 mg/24 h; 11–20 kg body weight, 50 mg/24 h; over 20 kg body weight 100 mg/24 h for prepubertal patients and 150 mg/24 h for pubertal patients) [48]. Alternatively, the following intravenous hydrocortisone doses have been recommended for major surgery or adrenal crisis: first, high dose bolus of hydrocortisone in an age-dependent manner: <1 year, 25 mg; 1–6 years, 50 mg; >6 years, 100 mg [46]. The hydrocortisone bolus must be followed by intravenous hydrocortisone for the next 24 h either as continuous infusion or frequent boluses: <1 year, 25–30 mg/24 h; 1–6 years, 50–60 mg/24 h; >6 years, 100 mg/24 h [46]. Alternatively, the following doses were recommended in children as follows: 50 mg/m^2^ as bolus followed by 50–100 mg during the first 24 h as continuous infusion or repeated boluses [47].

Hydrocortisone stress therapy can be progressively reduced 24–48 h after brain surgery depending on a patient’s general condition and pain control, and possibly stopped once the normal function of the hypothalamic–pituitary–adrenal axis is confirmed [30,47]. Hyponatremia in combination with hypoglycemia and hyperkalemia suggest adrenal crisis, and must be differentiated from hyponatremia due to the syndrome of inappropriate ADH secretion or cerebral salt wasting (see Table 2).

### 3.3. Central Hypothyroidism

Central hypothyroidism will not cause life-threatening post-surgery complications except in the context of coincident central hypocortisolism. Therefore, the assessment of the hypothalamic–pituitary-adrenal function is fundamental in patients presenting central hypothyroidism, as therapy with levothyroxine in case of not-substituted cortisol deficiency may lead to adrenal crisis by inducing cortisol metabolism and further reducing cortisol levels [49,50]. In the context of central hypocortisolism, levothyroxine should be started by increasing the dose over days while a full stress dose of hydrocortisone is given. Levothyroxine substitutive dose for patients with central hypothyroidism are as follows: 3.0–5.0 and 2.0–2.4 ug/kg/day during childhood and adolescence, respectively [49]. If in rare instances intravenous application is necessary, 80% of the oral dose of levothyroxine should be administered [51].

## 4. Late Endocrine Consequences according to Treatment Modalities

Childhood brain tumor survivors show a high-risk of endocrine deficits, predominantly of the hypothalamic–pituitary axis or the thyroid gland [52,53].

High-risk patients are those with central brain tumors after radiotherapy exposing the hypothalamus, pituitary, and thyroid, or after surgery near or within the hypothalamic–pituitary region.

The cumulative incidence of endocrine disorders in a cohort of *n* = 718 survivors of childhood brain tumors was 20.9% within the first five years of follow-up in one of the most comprehensive studies [54]. This cohort consisted of patients with different tumor types (low-grade gliomas 50%, medulloblastomas 14%, ependymomas 7%, and without or other histology 13% and 16%, respectively) and localizations (infratentorial 46%, supratentorial 38%, and central 16%) [54]. These patients were treated with different modalities (neurosurgery only 46%, neurosurgery and radiotherapy and chemotherapy 22%, neurosurgery and radiotherapy 13%, and neurosurgery and chemotherapy 7%) [54]. The prevalence of pituitary disorders was as follows: growth hormone (GH) deficiency 12%, precocious puberty 12%, thyroid-stimulating hormone (TSH) deficiency 9%, adrenocorticotropic hormone (ACTH) deficiency 4%, gonadotropin deficiency 4%, and ADH deficiency 3%. Prevalence of primary hypothyroidism and hypergonadotropic hypogonadism were 6% and 4%, respectively [54]. The risk for hypothalamic–pituitary deficiencies was associated with the following clinical parameters: male gender OR 1.5, hydrocephalus OR 1.8, tumor localization (suprasellar OR 34.2, infratentorial OR 2.6), and therapeutic parameters (neurosurgery OR 2.0, radiotherapy OR 15.7) [54].

In a recent follow-up study, younger age was associated with a significantly increased risk of hypothalamic–pituitary deficiencies, and non-irradiated infants and toddlers with brain tumor showed significantly increased TSH, ACTH, and ADH deficiencies compared to older brain tumor survivors [55].

Furthermore, the same authors identified in their cohort in *n* = 661 patients after a mean follow-up of 7.8 years a significant weight gain, overweight status, or obesity [22]. The prevalence of overweight and obese patients was 29% in childhood brain tumor survivors in comparison to 13% in the general pediatric population [22].

### 4.1. Post-Surgical Consequences

Brain surgery, per se, if not in the central region, does not seem to increase the risk of hypothalamic–pituitary dysfunctions according to a review of the literature for recent consensus guidelines [52], in contrast to surgery near or within the hypothalamic–pituitary region [54].

### 4.2. Post-Radiotherapy Consequences

Endocrine alterations after radiotherapy for brain tumors are well-documented [54,56,57,58,59]. They seem to be independent of the chosen technique (proton versus photon therapy) [52], although evidence from long-term data in large studies are missing. Recent data suggest that primary hypothyroidism might be less frequent in patients with medulloblastoma treated with craniospinal proton irradiation versus photon radiotherapy [60,61]. Furthermore, GH deficiency is the most frequent short-term and long-term post-radiotherapy endocrine disorder [53,54,58,62,63,64]. The risk of GH deficiency was 50% after 5 years of radiotherapy with 16 Gy [64] and may manifest in patients with either infratentorial or suprasellar tumors [54,62,63].

In a recent prospective study of *n* = 189 patients with a median follow up of 4.4 years, endocrine deficiencies were studied in response to different doses of proton radiotherapy [62]. It was found that 69% of patients with medulloblastoma were treated with craniospinal radiotherapy, while 14% with ependymoma and 7% with low-grade glioma were treated with cranial radiotherapy [62]. In the study, 16% of tumors were supratentorial and 84% were infratentorial [62]. The most frequent endocrine disorders according to the level of irradiation (<20 Gy, 20–40 Gy, and 40 Gy) were as follows: GH deficiency (9%, 40%, and 79%), followed by TSH deficiency (4%, 25%, and 43%), ACTH deficiency (4%, 4%, and 18%), and gonadotropin deficiency (0%, 3%, and 14%) [62]. In summary, this very comprehensive study provided detailed dose- and age-dependent incidences for each pituitary axis [62]. A recent study further detailed the incidences of endocrine deficiencies depending on the localization of brain tumors, with significantly more ACTH deficiency, hypothyroidism, and gonadotropin deficiency in suprasellar versus non-suprasellar tumors, but no difference in GH deficiency and precocious puberty [63].

Patients treated with craniospinal radiotherapy compared to those receiving only cranial irradiation may demonstrate a more altered adult final height [65] and a smaller upper/lower segment ratio [66].

### 4.3. Post-Chemotherapy Consequences

Platinum-based antineoplastic, alkylating agents, vinca alkaloids, etoposide, and methotrexate are frequently used in the treatment of pediatric brain tumors [67]. Patients treated only with chemotherapy demonstrate fewer late endocrine disorders than those receiving radiotherapy [68]. Moreover, chemotherapy in association with radiotherapy does not seem to increase the risk of alteration of the hypothalamic–pituitary axis, in particular GH and ACTH deficiencies, as well as of precocious puberty [52].

Typically, alkylating agents may damage the gonads, leading to premature ovarian insufficiency in women and impaired spermatogenesis or testosterone secretion in men [52,69].

### 4.4. Consequences of New-Therapeutic Agents

The use of immune modulators and protein kinase inhibitors has been recently described in the treatment of pediatric brain tumors, especially for low-grade gliomas, medulloblastomas, and ependymomas [5,67]. However, such agents may alter the thyroid function, leading to hypothyroidism and thyroiditis, and may have other severe side effects [70].

## 5. Late Endocrine Consequences and Their Management

Based on the current knowledge, the current guidelines [52] recommend the following clinical and biochemical screening measures starting one year after completion of radiotherapy or surgery near or within the hypothalamic–pituitary axis. As endocrine deficits can occur years after treatment, and often beyond pediatric age, these screening measures should be continued after transition to adult endocrinology or post-oncology care.

***Pre-pubertal child guidelines are as follows:***

▪ Clinical—height velocity, pubertal development;
▪ Biochemical—free thyroxin (FT4), TSH, and morning cortisol;
▪ Frequency—every six months in pre- and peri-pubertal patients;
▪ Warning signs—decline in growth velocity, precocious or delayed pubertal development, general clinical signs, and symptoms or laboratory tests suggestive of hypothalamic–pituitary deficiency.

***Adolescent and adult guidelines are as follows:***

▪ Clinical—general physical exam;
▪ Biochemical—FT4, TSH, morning cortisol, estradiol, follicle-stimulating hormone (FSH), and luteinizing hormone (LH) in females, morning testosterone and LH in males;
▪ Frequency—every 12 months in post-pubertal and adult patients;
▪ Warning signs—general clinical signs and symptoms or laboratory tests suggestive of hypothalamic–pituitary deficiency.

### 5.1. Central and Peripheral Hypothyroidism

Central hypothyroidism occurs typically in post high-dose irradiation patients with more than 30–40 Gy in the hypothalamic region (OR 12) and those treated with pituitary or hypothalamic surgery (OR 2.4). Central hypothyroidism is the third most frequent pituitary disorder (9.2%) observed in pediatric brain tumor survivors, after GH deficiency (12.2%) and precocious puberty (12.2%) [49,54].

Furthermore, patients may develop primary hypothyroidism after cranial or spinal irradiation or 131-I-metaiodobenzylguanidine (131-I-MIBG). Primary hypothyroidism may occur isolated or in combination with central hypothyroidism. The risk for peripheral hypothyroidism increases with a higher dose and longer time interval between treatment and follow-up, but cumulative incidence after 15 years of follow-up remained lower than central hypothyroidism in one large serie (5.8%) [54].

Clinical history and examination, as well as laboratory investigations, will confirm the diagnosis. Substitutive treatment with levothyroxine is indicated and needs to be titrated individually. Until adult height is not reached, more frequent controls of thyroid function are necessary, as thyroid hormones act in synergy with growth hormone for linear growth [71].

### 5.2. Thyroid Nodules and Secondary Differentiated Thyroid Carcinoma

The current guidelines [52], based on expert consensus and available evidence, recommend screening for thyroid nodules in patients after radiotherapy of the thyroid area and the use of therapeutic 131-I-MIBG. Despite the dose-dependent risk, all patients with thyroid radiation exposure even <1 Gy should be considered at risk for differentiated thyroid cancer, and surveillance should start at five years after radiotherapy or 131-I-MIBG [55,63,72,73,74].

One of the two following screening approaches is recommended after careful consideration of the advantages and disadvantages of each modality with the patient and his/her family:

1. Clinical screening—neck palpation (high specificity, but low sensitivity);
  Frequency—every 1–2 years;
2. Radiological screening—thyroid ultrasound (high specificity and sensitivity);
  Frequency—every 3–5 years.

In our experience at both centers, the combination of both modalities is well accepted by patients and families. Therefore, we suggest performing clinical and radiological screening at least every 1–2 years.

### 5.3. GH Deficiency

Children and adolescents after brain tumor treatment should be followed carefully for pathologic growth and decreased growth velocity [52]. Decreased growth may not only be due to GH deficiency, but other endocrine deficits (central or peripheral hypothyroidism and central or peripheral hypogonadism), and non-endocrine reasons (post-radiation impairment of the growth plate in the spine, possibly resulting in disproportional growth of the spine versus the long bones of arms and legs) [53]. Nevertheless, GH deficiency needs to be excluded in all pediatric cancer survivors with poor linear growth until reaching adult height, sometimes even several times during the follow-up. Therefore, auxology is crucial for the early detection of GH deficiency. Adult growth hormone deficiency should not be ignored and retesting in adult pediatric cancer survivors with GH deficiency is recommended [53].

Diagnosis of GH deficiency may be challenging in patients treated for brain tumors due to alteration of the hypothalamus [75,76]. Differently to the general population, IGF1 and IGFBP3 do not seem to be reliable markers [75]. Among all the available provocative tests, the insulin tolerance test appears to be the most effective [75].

A recent consensus statement reviewed all aspects of safety of GH replacement therapy in survivors of cancer and intracranial and pituitary tumors [76]. The GH substitution is in general possible after at least 1 year of observation after the end of the therapy, only in patients with radiologically stable disease over 12 months, and is not associated with higher risk of recurrence of the primary tumor [76,77,78,79]. In specific clinical situations, a longer interval between the end of therapy and the start of GH substitution might be necessary, and need to be individualized.

### 5.4. Delayed Puberty and Disorders of Gonadal Function

In pediatric brain tumor survivors, delayed puberty may be a consequence of central (or hypogonadotropic hypogonadism) due to pituitary or hypothalamic surgery or irradiation, or from peripheral (or hypergonadotropic hypogonadism) mostly as a sequela of gonadotoxic chemotherapy [80,81].

In girls, delayed puberty is defined by the absence of pubertal signs (thelarche) or primary amenorrhea by the age of 13 or 16 years, respectively [52]. Premature ovarian failure is characterized by the absence of ≥4 menstrual cycles in women <40 years or by an arrest of pubertal development in girls ≥13 years [52,82]. Two increased FSH values in the menopausal range are suggestive of premature ovarian failure, while non-measurable gonadotropins are indicative of hypogonadotropic hypogonadism [52]. Anti-Müllerian hormone (AMH) is an indirect marker of the ovarian reserve [83]. However, AMH may be only used in association with FSH and estradiol in female cancer survivors due to missing data [84].

In boys, delayed puberty is defined by the lack of pubertal signs (increase in testicular volume) by the age of 14 years [52]. As for girls, non-measurable gonadotropins are suggestive of hypogonadotropic hypogonadism. In boys with arrested pubertal development, low morning testosterone and/or increased LH are suggestive of gonadal failure [52].

In case of doubt in first-line blood tests, second-line investigations, such as the GnRH-test, may be helpful to differentiate between central or peripheral hypogonadism [85,86].

Standard puberty induction schemas and estradiol/testosterone substitution in case of gonadal dysfunction are outlined in Table 3. A timely substitutive therapy in pediatric brain tumor survivors with delayed puberty is crucial not only for quality of life and psychosocial well-being, but for optimal bone mineralization in this population at risk of decreased bone mineral density (see Section 5.7 below).

Fertility preservation in pediatric cancer patients has become feasible under certain conditions. Oocytes and sperm preservation are possible in adolescents before the initiation of potential gonadotoxic agents [52,69]. Cryopreservation of gonadal tissue may be sometimes possible under certain conditions [83].

### 5.5. Precocious Puberty

Precocious puberty is defined by the appearance of typical puberty signs before eight years of age in girls and before nine years of age in boys. Precocious puberty is a consequence of premature activation of the hypothalamic–pituitary–gonadal axis with increased LH and FSH resulting in ovarian or testicular stimulation, growth, and estrogen or testosterone production. Pediatric brain tumor survivors at risk are those with a cranial irradiation of more than 18 Gy (OR 3.0), hydrocephalus (OR 3.7), and tumors of the hypothalamic and pituitary region (OR 110). Female sex and a younger age are further risk factors [54,91,92].

Treatment of these patients with GnRH agonists to suppress the premature activation of the gonadal axis until adequate age for puberty is performed in analogy with patients with idiopathic precocious puberty [53].

### 5.6. Central Diabetes Insipidus

Central diabetes insipidus may be present at diagnosis or appear after surgery. It is the least frequent endocrine deficiency occurring in the late follow-up, with a cumulative incidence of 2.6% at 15 years [54]. Diabetes insipidus rarely occurs after cranial irradiation [93,94]. For treatment with desmopressin, see Section 3.1 above.

### 5.7. Bone Fragility

A Z-score <−2 SD in bone densitometry is suggestive of osteoporosis in children and adolescents, but only in association with a history of bone fractures (either ≥2 long bone fractures in patients under 10 years or ≥3 long bone fractures above 10 years) [69,95]. However, in patients with risk factors for bone fragility (e.g., therapy with glucocorticoids, hypogonadism, and premature ovarian failure), a long bone fracture consecutive to low-trauma (e.g., fall from a standing height or less) is also suggestive for osteoporosis [95,96]. More importantly, vertebral fractures alone, even without bone mineral density alteration, are always suggestive of osteoporosis [69,95].

In girls ≥8 years and boys ≥9 years with such risk factors, spine imaging should be performed annually as vertebral fractures are often asymptomatic, or immediately in case of back pain or two consecutive bone densitometry tests with a Z-score decline of ≥0.5 [95]. Bone densitometry is routinely performed once a year [95].

In children or adolescents with secondary osteoporosis, the indication to therapy with intravenous bisphosphonates depends on the underlaying cause (transient versus persistent) [97]. Cranial or spinal irradiation (≥24 Gy) are correlated with poor spontaneous remineralization [98].

In addition to hypogonadism and irradiation, two recent cohort studies showed that decreased bone mineral density in pediatric brain tumor survivors is associated with inadequate calcium and vitamin D intake, vitamin D deficiency, and an inactive lifestyle [99,100].

### 5.8. Hypothalamic Obesity

Hypothalamic syndrome or hypothalamic obesity is a severe disorder occurring in patients with hypothalamic injury due to the tumor itself or as a sequela of its treatment. The clinical consequences of hypothalamic damage encompass psychosocial disorders, hyperphagia due to damage of the appetite regulation by resistance of satiety hormones, such as insulin and leptin, sleep disturbances, decreased energy expenditure, hyperinsulinism, and hypopituitarism [20,101]. Morbid obesity with all metabolic consequences is a part of hypothalamic syndrome, in combination with neurocognitive symptoms [20]. Its incidence decreased significantly after a change from total to subtotal pituitary resection in patients with craniopharyngioma [102].

## 6. Conclusions

Endocrine deficits may be present at or even before the diagnosis of pediatric brain tumors, may occur in the acute post-surgical period, and are highly prevalent during the long-term follow-up in survivors. Altered auxologic parameters, clinical signs, and symptoms of hypothalamic–pituitary deficiencies, together with neurological or neuroophthalmological signs, should raise suspicion of brain tumors at any age and lead to prompt investigations. During the post-surgical period, disorders of water homeostasis are frequent. Close observation of diuresis, serum sodium, and urine osmolality allow us to detect and differentiate diabetes insipidus, the syndrome of inappropriate ADH secretion, and cerebral salt wasting. Systematic screening of endocrine deficiencies is standard in pediatric brain tumor survivors with detailed new age-dependent recommendations. Early detection and substitution of specific deficits result in improved general and metabolic health and quality of life.

## Figures and Tables

**Table 1:** Typical symptoms for each age group for which care givers and medical personal should seek medical evaluation, adapted from www.headsmart.org.uk (accessed on 8 July 2022) [16,17,18].

		Age		
		<5 Years	5–12 Years	12–18 Years
Neurological and visual symptoms	Persistent or recurrent vomiting			
	Balance, co-ordination, walking problems			
	Abnormal eye movements			
	Suspected loss of vision			
	Blurred or double vision, loss of vision			
	Behavior change			
	Seizures or fits (without fever)			
	Abnormal head position			
	Reduced consciousness			
	Fastly increasing head circumference			
	Persistent or recurrent headache			
Endocrine symptoms	Diabetes insipidus			
	Abnormal growth			
	*Early puberty* ^a,b^			
	Delayed or suspended puberty ^b^			
Seek for specific medical evaluation if the following are true: - ≥2 symptoms - Symptoms persist for ≥2 weeks		Seek for emergency evaluation if the following are true: - Symptoms are severe - Symptoms develop quickly		

Note: Grey = symptom may be present; ^a^ Added by the authors; ^b^ See Section 5.4 and Section 5.5 for definitions.

**Table 2:** Differential diagnosis of post-operative water homeostasis alterations [33,34,35,37].

	Diagnosis	Clinical Features			Therapy
		Fluid Status	Diuresis	Vital Parameters	
*Hypernatremia*	Diabetes insipidus	Euvolemic	Polyuria	If thirst mechanism intact and/or normal ability to drink: HR normal, BP normal	Free access to oral fluid
		Hypovolemic	Polyuria	If patient adipsic or not able to drink: HR high, BP low	Fluid replacement, desmopressin
*Hyponatremia*	Syndrome of inappropriate ADH secretion	Euvolemic	Oliguria	HR normal, BP normal	Fluid restriction
	Cerebral salt wasting	Hypovolemic	Polyuria	HR high, BP low	Fluid and sodium replacement
	Adrenal crisis	Hypovolemic	Polyuria	HR high, BP low	Hydrocortisone, and fluid and sodium replacement

Note: HR = heart rate; BP = blood pressure.

**Table 3:** Puberty induction and substitution therapy in case of gonadal dysfunction [87,88,89,90].

	Therapy
Girls	Estradiol transdermal patches [87]: - Tanner 1: 0.06 ug/kg overnight - Tanner 2: 0.1 ug/kg overnight - Tanner 3: 0.2 ug/kg overnight - Tanner 3–4: 0.35 ug/kg overnight and half that dose during the day - Tanner 4–5: 0.8 ug/kg 2–3 days each week Add progestin per os after 12–18 months of estradiol [88,89] or after menarche: - 5–10 mg daily for 10 days every period [87] In young adults, evaluate combined pills [87].
Boys	Testosterone esters intramuscular: - 50 mg each 4 weeks up to 6 months for puberty induction, eventually increase up to 100 mg each 4 weeks [88,89] - If long-term substitution required, increase progressively over 3 years up to adult dose of 250 mg each every 4 weeks [88,89,90]

## Data Availability

Not applicable.
